# Supplementary material for: The Small RNA Universe of Capitella teleta
Source: Front Mol Biosci. 2022 Feb 25;9:802814. doi: 10.3389/fmolb.2022.802814 (PMC8915122; doi:10.3389/fmolb.2022.802814)
Supplement: Supplementary file 1 [file DataSheet1.ZIP › Supplement/known/CAPTEscaffold_274_15674.pdf]

Provisional ID	:	CAPTEscaffold_274_15674
Score total	:	18614.9
Score for star read(s)	:	3.9
Score for read counts	:	18605.2
Score for mfe	:	1.3
Score for randfold	:	1.6
Score for cons. seed	:	3
Total read count	:	36505
Mature read count	:	33861
Loop read count	:	23
Star read count	:	2621

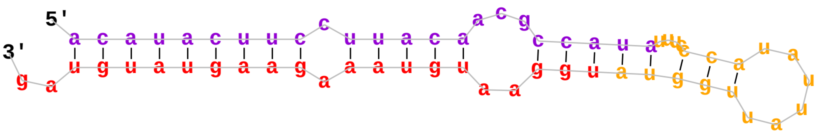

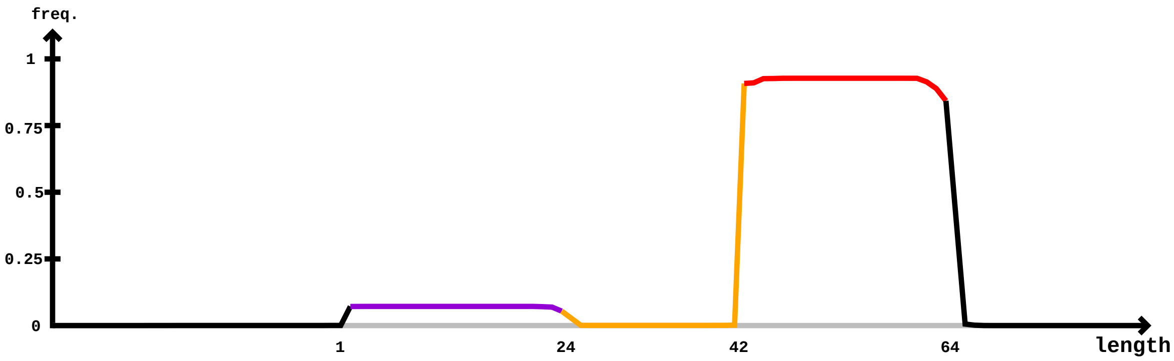


```
                                                Star                                      Mature
5'- gcugcaagaacuaccggcgugcgauugugacauacuuccuuacaacgccauauuucccauauuauugguauggaauguaaagaaguauguagcauucccaugcacuuuuuca  -3'   obs
    gcugcaagaacuaccggcgugcgauugugacauacuuccuuacaacgccauauuucccauauuauugguauggaauguaaagaaguauguagcauucccaugcacuuuuuca         exp
    gcugcaagaacuaccggcgugcgauugugacauacuuccuuacaacgccauauuucccauauuauugguauggaauguaaagaaguauguagcauucccaugcacuuuuuca         known
    .......(((......(((((.((.(((.(((((((((.(((((...(((((....(((......))))))))..))))).))))))))).))).)).))))).....))).   reads   mm   sample
    ......agaacuaccggcgugcgauugug...................................................................................   1       0    seq
    ........aacuaccggcgugcgauugug...................................................................................   1       0    seq
    ..........cuaccggcgugcgauugug...................................................................................   3       0    seq
    ...........uaccggcgugcgauugug...................................................................................   2       0    seq
    ...........................ugacauacuuccuuacaacgcc...............................................................   1       0    seq
    ...........................ugacauacuuccuuacaacgccU..............................................................   1       1    seq
    ...........................ugacauacuuccuuacaacgcca..............................................................   4       0    seq
    ...........................uCacauacuuccuuacaacgccau.............................................................   2       1    seq
    ............................gacauacuuccuuacaacgccau.............................................................   1       0    seq
    .............................acauacuuccuuacaacgcc...............................................................   33      0    seq
    .............................acauacuuccuuacaacgcca..............................................................   56      0    seq
    .............................acauacuucUuuacaacgccau.............................................................   1       1    seq
    .............................acauacuuccuuacaacgccau.............................................................   532     0    seq
    .............................Gcauacuuccuuacaacgccau.............................................................   4       1    seq
    .............................acauacuuccuuacaacgcAau.............................................................   1       1    seq
    .............................aAauacuuccuuacaacgccau.............................................................   2       1    seq
    .............................acauaAuuccuuacaacgccau.............................................................   1       1    seq
    .............................acauacuucAuuacaacgccau.............................................................   1       1    seq
    .............................acauacuuccuuacaacgccaua............................................................   1820    0    seq
    .............................aAauacuuccuuacaacgccaua............................................................   1       1    seq
    .............................acauacuuccuuacaacgcAaua............................................................   2       1    seq
    .............................acaAacuuccuuacaacgccaua............................................................   2       1    seq
    .............................Ncauacuuccuuacaacgccaua............................................................   1       1    seq
    .............................acauacuuccuuacaacgcUaua............................................................   1       1    seq
    .............................acauacAuccuuacaacgccaua............................................................   4       1    seq
    .............................acauacuuccuuacaacgccauU............................................................   77      1    seq
    .............................acauacuuccuuacaacgccauG............................................................   1       1    seq
    .............................acauacuuccuCacaacgccaua............................................................   1       1    seq
    .............................acauaAuuccuuacaacgccaua............................................................   2       1    seq
    .............................acauacuuccuuacaacgccUua............................................................   1       1    seq
    .............................acauacuAccuuacaacgccaua............................................................   1       1    seq
    .............................acauacuuccuAacaacgccaua............................................................   1       1    seq
```

```
                             Star                                    Mature
gcugcaagaacuaccggcgugcgauugugacauacuuccuuacaacgccauauuucccauauuauugguauggaauguaaagaaguauguagcauucccaugcacuuuuuca
.............................Gcauacuuccuuacaacgccaua............................................................   25    1   seq
.............................acauacuuccuuacaGcgccaua............................................................   1     1   seq
.............................acauacuucAuuacaacgccaua............................................................   1     1   seq
.............................acauacuucUuuacaacgccaua............................................................   2     1   seq
.............................acauacuuccuuacaacgccauUu...........................................................   9     1   seq
.............................acauacuuccuuacaacgccauau...........................................................   10    0   seq
.............................Gcauacuuccuuacaacgccauau...........................................................   1     1   seq
.............................acauacuuccuuacaacgccauaA...........................................................   4     1   seq
.............................acauacuuccuuacaacgccauUuu..........................................................   2     1   seq
.............................acauacuuccuuacaacgccauauC..........................................................   1     1   seq
..............................cauacuuccuuacaacgccau.............................................................   4     0   seq
..............................cauacuuccuuacaacgccaua............................................................   4     0   seq
..............................cauacuuccuuacaacgccauau...........................................................   1     0   seq
...............................aCacuuccuuacaacgccaua............................................................   1     1   seq
..................................................uauuucccauauuauugguaug........................................   1     0   seq
....................................................uuucccauauuauuggua..........................................   22    0   seq
...................................................................guauggaauguaaagaaguaug.......................   3     0   seq
....................................................................uauggaauguaaagaaguaugu......................   1     0   seq
....................................................................uCuggaauguaaagaaguauguag....................   2     1   seq
.....................................................................Guggaauguaaagaaguaugu......................   4     1   seq
.....................................................................Uuggaauguaaagaaguaugua.....................   2     1   seq
.....................................................................auggaauguaaagaaguaugua.....................   4     0   seq
.....................................................................auggaauguaaagaaguauguag....................   12    0   seq
.....................................................................Cuggaauguaaagaaguauguag....................   2     1   seq
.....................................................................auggaauguaaagCaguauguag....................   1     1   seq
......................................................................uggUauguaaagaaguau........................   1     1   seq
......................................................................uggaauguaaagaaguau........................   7     0   seq
......................................................................Gggaauguaaagaaguau........................   1     1   seq
......................................................................uggUauguaaagaaguaug.......................   1     1   seq
......................................................................uggaauAuaaagaaguaug.......................   1     1   seq
......................................................................Aggaauguaaagaaguaug.......................   3     1   seq
......................................................................uggaauguaaagaaguauA.......................   1     1   seq
......................................................................uggaauguaaagaaguauU.......................   12    1   seq
......................................................................uggaauguaaagaaguaug.......................   447   0   seq
......................................................................ugCaauguaaagaaguaug.......................   1     1   seq
......................................................................uggaGuguaaagaaguaug.......................   17    1   seq
......................................................................uggaauguaaagaaguGug.......................   1     1   seq
......................................................................uAgaauguaaagaaguaug.......................   4     1   seq
......................................................................Gggaauguaaagaaguaug.......................   1     1   seq
......................................................................Gggaauguaaagaaguaugu......................   1     1   seq
......................................................................Cggaauguaaagaaguaugu......................   1     1   seq
......................................................................uggaauguaaagaaguaugu......................   888   0   seq
......................................................................uggaauguaaagGaguaugu......................   1     1   seq
......................................................................uggaauguaaagaaguaugG......................   1     1   seq
......................................................................Aggaauguaaagaaguaugu......................   4     1   seq
......................................................................ugAaauguaaagaaguaugu......................   2     1   seq
......................................................................uggaGuguaaagaaguaugu......................   1     1   seq
......................................................................ugUaauguaaagaaguaugu......................   1     1   seq
......................................................................uggaaugCaaagaaguaugu......................   1     1   seq
......................................................................uggaauguaaagaaguaugA......................   2     1   seq
......................................................................uAgaauguaaagaaguaugu......................   2     1   seq
......................................................................uggaauguaaagaaguaGgu......................   1     1   seq
......................................................................uggaGuguaaagaaguaugua.....................   10    1   seq
......................................................................uAgaauguaaagaaguaugua.....................   2     1   seq
......................................................................uggaauguaaagaaguauguU.....................   9     1   seq
......................................................................uggaauguaaGgaaguaugua.....................   2     1   seq
......................................................................ugAaauguaaagaaguaugua.....................   1     1   seq
......................................................................uggUauguaaagaaguaugua.....................   1     1   seq
......................................................................uggaauguaaagaGguaugua.....................   1     1   seq
......................................................................Aggaauguaaagaaguaugua.....................   13    1   seq
......................................................................uggGauguaaagaaguaugua.....................   1     1   seq
......................................................................uggaauguaaagaaguaugua.....................   1626  0   seq
......................................................................uggaauguaaagaaguGugua.....................   1     1   seq
......................................................................uggaauguGaagaaguaugua.....................   1     1   seq
......................................................................ugCaauguaaagaaguaugua.....................   2     1   seq
......................................................................uCgaauguaaagaaguaugua.....................   1     1   seq
......................................................................uggaauguaaagaaAuauguag....................   2     1   seq
......................................................................uggaauguaaaAaaguauguag....................   2     1   seq
......................................................................uggaauguaaagaaguaAguag....................   4     1   seq
......................................................................uggaUuguaaagaaguauguag....................   3     1   seq
```

```
                             Star                                     Mature
gcugcaagaacuaccggcgugcgauugugacauacuuccuuacaacgccauauuucccauauuauugguauggaauguaaagaaguauguagcauucccaugcacuuuuuca
......................................................................uggaauguaaagaaguauguag....................  21803  0  seq
......................................................................uggaauguaaagaaUuauguag....................  2      1  seq
......................................................................uggaauguaaUgaaguauguag....................  1      1  seq
......................................................................uggaauguaaaUaaguauguag....................  2      1  seq
......................................................................uCgaauguaaagaaguauguag....................  8      1  seq
......................................................................uggaauguaaagaagGauguag....................  4      1  seq
......................................................................Aggaauguaaagaaguauguag....................  120    1  seq
......................................................................uggaauguaGagaaguauguag....................  4      1  seq
......................................................................uggaauguaaagaaguauguUg....................  3      1  seq
......................................................................uggGauguaaagaaguauguag....................  5      1  seq
......................................................................ugCaauguaaagaaguauguag....................  32     1  seq
......................................................................uggaauguaaagaaguauAuag....................  3      1  seq
......................................................................uggaGuguaaagaaguauguag....................  87     1  seq
......................................................................uggaaAguaaagaaguauguag....................  6      1  seq
......................................................................uggaauguaaagaaguaGguag....................  2      1  seq
......................................................................uggUauguaaagaaguauguag....................  2      1  seq
......................................................................uggaauguaaGgaaguauguag....................  20     1  seq
......................................................................uggaauguaaagaaguaugGag....................  1      1  seq
......................................................................uggaauCuaaagaaguauguag....................  1      1  seq
......................................................................uggaauguaaagUaguauguag....................  2      1  seq
......................................................................uggaaugGaaagaaguauguag....................  1      1  seq
......................................................................uggaauguaaagaaguauCuag....................  1      1  seq
......................................................................uggaauAuaaagaaguauguag....................  2      1  seq
......................................................................uggaauguaaagaaguauUuag....................  2      1  seq
......................................................................uggaauguaaagaaguUuguag....................  3      1  seq
......................................................................uggaaugAaaagaaguauguag....................  12     1  seq
......................................................................uggaauguaaagaaguauguaU....................  124    1  seq
......................................................................uggaauguaaagGaguauguag....................  3      1  seq
......................................................................Gggaauguaaagaaguauguag....................  27     1  seq
......................................................................uggaauUuaaagaaguauguag....................  4      1  seq
......................................................................uggaauguaUagaaguauguag....................  1      1  seq
......................................................................uggaauguaaagaaguauguGg....................  11     1  seq
......................................................................uggaauguaaagaaguGuguag....................  3      1  seq
......................................................................uggaauguGaagaaguauguag....................  5      1  seq
......................................................................uggaaugCaaagaaguauguag....................  1      1  seq
......................................................................uggaauguaaagaagAauguag....................  9      1  seq
......................................................................uggaaCguaaagaaguauguag....................  2      1  seq
......................................................................uggaauguaaagaaCuauguag....................  2      1  seq
......................................................................Nggaauguaaagaaguauguag....................  6      1  seq
......................................................................ugAaauguaaagaaguauguag....................  9      1  seq
......................................................................uggaauguaaagaaguauguCg....................  1      1  seq
......................................................................uggaauguaaagaaguaugCag....................  2      1  seq
......................................................................Cggaauguaaagaaguauguag....................  10     1  seq
......................................................................uggaauguaaagaGguauguag....................  6      1  seq
......................................................................uggaauguaaagaaguauguaA....................  72     1  seq
......................................................................uUgaauguaaagaaguauguag....................  5      1  seq
......................................................................uggaauguaaagaaguaugAag....................  9      1  seq
......................................................................uAgaauguaaagaaguauguag....................  74     1  seq
......................................................................uggaauguaaagaaguauguaC....................  199    1  seq
......................................................................uggaauguaaagaUguauguag....................  2      1  seq
......................................................................uggaauguaaagaaguauguaCc...................  1      1  seq
......................................................................uggaauguaaagaaguauguagG...................  2      1  seq
......................................................................uggaauguaaagaaguUuguagc...................  1      1  seq
......................................................................uAgaauguaaagaaguauguagc...................  2      1  seq
......................................................................uggaauguaaagaaguauguagc...................  21     0  seq
......................................................................uggaauguaaagaaguauguagA...................  1379   1  seq
......................................................................uggaauguaaagaaguauguagU...................  5790   1  seq
......................................................................Aggaauguaaagaaguauguagc...................  1      1  seq
......................................................................uggaauguaaagaaguauguagUa..................  31     1  seq
......................................................................uggaauguaaagaaguauguagAa..................  85     1  seq
......................................................................uggaauguaaagaaguauguagGa..................  1      1  seq
.......................................................................Ugaauguaaagaaguaugua.....................  1      1  seq
.......................................................................ggaauguaaagaaguaugua.....................  1      0  seq
.......................................................................ggaauguaaagaaguauguag....................  2      0  seq
.......................................................................Ugaauguaaagaaguauguag....................  5      1  seq
.......................................................................ggaauguaaagaaguauguagA...................  17     1  seq
.......................................................................ggaauguaaagaaguauguagc...................  1      0  seq
.......................................................................ggaauguaaagaaguauguagU...................  60     1  seq
.......................................................................ggaauguaaagaaguauguagAa..................  1      1  seq
........................................................................gaauguaaagaaguauguag....................  2      0  seq
```

```
                              Star                                      Mature
gcugcaagaacuaccggcgugcgauugugacauacuuccuuacaacgccauauuucccauauuauugguauggaauguaaagaaguauguagcauucccaugcacuuuuuca
........................................................................gaauguaaagaaguauguagc...................    43     0     seq
........................................................................gaauguaaagaaguauguagU...................    252    1     seq
........................................................................gaauguaaagaaguauguaCc...................    2      1     seq
........................................................................gaauguaaagaaguauguagA...................    189    1     seq
........................................................................Aaauguaaagaaguauguagc...................    1      1     seq
........................................................................gaauguaaagaaguauguagca..................    1      0     seq
........................................................................gaauguaaagaaguauguagAa..................    9      1     seq
........................................................................gaauguaaagaaguauguagUa..................    28     1     seq
........................................................................gaauguaaagaaguauguagAau.................    12     1     seq
........................................................................gaauguaaagaaguauguagUau.................    35     1     seq
........................................................................gaauguaaagaaguauguagUauu................    2      1     seq
.........................................................................aauguaaagaaguaugua.....................    2      0     seq
.........................................................................aauguaaagaaguauguag....................    7      0     seq
.........................................................................aauguaaagaaguauguagU...................    6      1     seq
..........................................................................auguaaagaaguauguag....................    27     0     seq
..........................................................................auguaaagaaguauguagA...................    9      1     seq
...........................................................................uguaaagaaguauguagA...................    2      1     seq
............................................................................................cauucccaugcacuuuuuc.    1      0     seq
```
